# Improved biodiesel production from waste cooking oil with mixed methanol–ethanol using enhanced eggshell-derived CaO nano-catalyst

**DOI:** 10.1038/s41598-021-86062-z

**Published:** 2021-03-23

**Authors:** Yeshimebet Simeon Erchamo, Tadios Tesfaye Mamo, Getachew Adam Workneh, Yedilfana Setarge Mekonnen

**Affiliations:** 1 Center for Environmental Science, College of Natural and Computational Sciences, Addis Ababa University, P. O. Box 1176, Addis Ababa, Ethiopia; 2 Ethiopia Chemical and Construction Inputs Industry Development Institute, P. O. Box 4124, Addis Ababa, Ethiopia; 3 Center of Excellence in Sustainable Energy, Department of Industrial Chemistry, College of Applied Science, Addis Ababa Science and Technology University, P. O. Box 16417, Addis Ababa, Ethiopia; 4 Ministry of Mine and Petroleum, Federal Democratic Republic of Ethiopia, P. O. Box 486, Addis Ababa, Ethiopia

**Keywords:** Environmental sciences, Chemistry, Energy science and technology, Materials science, Nanoscience and technology

## Abstract

In this report, the utilization of mixed methanol–ethanol system for the production of biodiesel from waste cooking oil (WCO) using enhanced eggshell-derived calcium oxide (CaO) nano-catalyst was investigated. CaO nano-catalyst was produced by calcination of eggshell powder at 900 °C and followed by hydration-dehydration treatment to improve its catalytic activity. The particle size, morphology, and elemental composition of a catalyst were characterized by using XRD, SEM, and EDX techniques, respectively. After hydration-dehydration the shape of a catalyst was changed from a rod-like to honeycomb-like porous microstructure. Likewise, average particle size was reduced from 21.30 to 13.53 nm, as a result, its surface area increases. The main factors affecting the biodiesel yield were investigated, accordingly, an optimal biodiesel yield of 94% was obtained at 1:12 oil to methanol molar ratio, 2.5 wt% catalyst loading, 60 °C, and 120-min reaction time. A biodiesel yield of 88% was obtained using 6:6 equimolar ratio of methanol to ethanol, the yield even increased to 91% by increasing the catalyst loading to 3.5 wt%. Moreover, by slightly increasing the share of methanol in the mixture, at 8:4 ratio, the maximum biodiesel yield could reach 92%. Therefore, we suggest the utilization of methanol–ethanol mixture as a reactant and eggshell-derived CaO as a catalyst for enhanced conversion of WCO into biodiesel. It is a very promising approach for the development of low-cost and environmentally friendly technology. Properties of the biodiesel were also found in good agreement with the American (ASTM D6571) fuel standards.

## Introduction

Energy is among the key pillars for one’s country’s economic and social development. Currently, lion shares of the world energy demand still supplied through non-renewable sources, which accounts for about 78% of the total share. Nowadays they are being consumed very quickly and have limited reserves^1^. Immense carbon dioxide (CO_2_) and greenhouse gas emissions (GHGs) from different anthropogenic activities trigger environmental pollution, environmental deterioration, and global warming^2,3^. Moreover, these trace gases are also responsible for climate change. Consequently, reducing these emissions is critical, this can be achieved by focusing on the mitigation strategies including the utilization of renewable energy systems. Searching for an alternative non-fossil fuel-based energy system that is renewable, sustainable, economical, and environmentally benign is inevitable. Biodiesel is a liquid biofuel explored as a conventional mitigation technology with low-carbon emissions. Therefore, substituting petroleum fuels with biodiesel could be a plausible means to mitigate CO_2_ and other GHGs emissions particularly in the transportation sector, and thus, it plays a vital role in reducing environmental related problems, global warming, and climate change^4^.

Biodiesel is a renewable, clean-burning liquid fuel, which can be produced by transesterification reaction from biomass sources such as vegetable oil, animal oil/fat, and waste cooking oil^2,5,6^. The main problem facing biodiesel commercialization and its market competitiveness is its production cost and the expensiveness of the raw materials used in the production of biodiesel^2,6–8^. Compared to petrol diesel, biodiesel from common foodstuff is more expensive, and hence to tackle this problem it is vital to focus on searching for economical and readily available feedstocks, alcohols, and catalysts. Biodiesel production from waste such as waste cooking oil (WCO) and eggshell-derived calcium oxide (CaO) is a promising alternative for the production of low-cost and environmentally sustainable products^5–12^. Using waste cooking oil and eggshell for biodiesel production has a dual advantage which is an effective way to reduce the production cost of biodiesel and reduce environmental pollution problems due to the disposal of waste cooking oil and eggshell.

CaO is the most widely used heterogeneous base catalyst naturally abundant as limestone. In addition to limestone, there are other effective sustainable natural sources of CaO such as crab shells, eggshells, capiz shells, abalone shells, snail shells, oyster shells, mussel shells, and goat bone^2,10,11,13–19^. Besides, apart from CaO-based catalyst other catalysts such as MgO^20^, Mg/Al hydrotalcite^21–24^, nano-magnetic catalyst KF/CaO–Fe_3_O_4_^25^, hydrated lime-derived CaO^26^, and CaO–Fe_2_O_3_^27^ have been explored for biodiesel production. Transesterification is the reaction of a fat or oil triglyceride with an alcohol to form esters and glycerol which is a reversible process and thus an excess of alcohol is required to force the equilibrium to the product side. Theoretically, the stoichiometry for the transesterification reaction is 3:1 alcohol to oil, however, in practice, the amount can vary, as it is experimentally determined. Several alcohols have been studied for biodiesel production of which methanol and ethanol are the two most frequently used in the production of biodiesel^8,12,28–30^. Using methanol or ethanol has its advantage and disadvantage related to the physical and chemical properties of the transesterification reaction and its product.

Methanol is the most preferred alcohol chosen due to reactivity and high equilibrium conversion, but it is a toxic fossil fuel-based product and exhibits mass transfer limitations in transesterification reaction due to its limited solubility in oil^31^. On the other hand, transesterification using ethanol has limitations due to the formation of stable emulsion and difficulty in separation of biodiesel and glycerol associated with high solubility and less reactivity of ethanol^32^. The main advantage of using ethanol is; it can be obtained from a renewable source and is not toxic.

Several previous works have reported on the utilization of CaO material as a catalyst in the transesterification reaction from different sources. CaO catalyst is prepared from natural sources, mostly from different waste shells. As stated by Rezaei et al. (2013), a biodiesel yield of 94.1% was obtained from soybean oil using CaO catalyst prepared from mussel shell with pronounced purity at a specified experimental setting^18^. Likewise, Chen et al. (2014) also reported a 93% biodiesel yield from palm oil using CaO catalyst made from ostrich eggshell prepared through an ultrasonic technique^33^. Moreover, Chouhan et al. (2011) achieved a biodiesel yield of more than 97% from soybean oil using an actual CaO catalyst prepared from chicken waste eggshell at optimal experimental conditions. They demonstrated the practicality of waste chicken eggshell-based CaO catalyst for high yield and good quality biodiesel production^34^. Similarly, Gupta et al. (2016) also reported a biodiesel yield of 96% from soybean oil using snail eggshell-based CaO catalyst at a particular experimental condition^35^.

As witnessed from several previous studies, the CaO catalyst obtained from the natural source has effectively catalyzed the transesterification of high-grade or virgin vegetable oil. Nevertheless, consuming high-grade or unused vegetable oil for biodiesel production is not economical. This is one of the critical challenges that impede entering into the biodiesel market. Towards overwhelmed this constraint many research activities are carried-out for searching an alternative feedstock that is renewable, environmentally friendly, sustainable, and economical. In this regard, the utilization of waste cooking oil and a variety of non-edible plant oils such as jatropha, castor, linseed, and tobacco for the transesterification reaction are important feedstock. Even if the use of waste cooking oil for biodiesel production has been well explored in several previous works, there are still some shortcomings such as the existence of high free fatty acid content and impurities. These limitations must be overcome or reduce for it to be considered as a promising candidate for conventional homogenously-catalyzed biodiesel production^36^. In conjunction, different studies were conducted on biodiesel production from waste cooking oil by using eggshell catalysts and they confirmed an optimal yield of 91%^11^ and 100%^37^ at different reaction conditions. Unlike homogenous catalyzed transesterification, the heterogeneously-catalyzed transesterification reactions were relatively taken longer reaction time. This happened due to the mixing problem of the three-phases systems (oil-alcohol-catalyst)^38^. The low basicity and surface area of a CaO potentially limit its catalytic activity. As a consequence, higher catalyst loading and large reaction time might be required for the transesterification process to complete, which encounters an additional cost of production. The catalytic activity of CaO can be improved for an enhanced biodiesel yield by increasing the basicity and surface area of a catalyst. The latter can be achieved by reducing a crystal size, for example synthesizing a nanoparticle can remarkably increase the catalytic performance of a heterogeneous catalyst. Hence, to alleviate this problem reducing the heterogeneous catalyst size is one key step.

Currently, several studies have been conducted to improve the catalytic activity of CaO with different techniques relevant for transesterification reaction. Yoosuk et al. (2010) reported that the decomposed hydrated CaO exhibited higher catalytic activity even at shorter reaction time compared to CaO produced from direct calcination of CaCO_3_ under mild reaction conditions. About 93.9% biodiesel yield was accomplished at oil to methanol molar ratio 1:15, catalyst loading 7 wt%, reaction temperature 60 °C, and a reaction time of an hour. Interestingly, high biodiesel yield was achieved within an hour of reaction time mainly due to an improvement of catalytic activity of hydrated CaO catalysts^39^. Moreover, Asikin-Mijan et al. (2015) showed that hydration treated clam shell derived CaO effectively convert palm oil into biodiesel with 98% yield after 2 h of reaction time at methanol to oil molar ratio of 9:1, catalyst loading of 1wt%, and temperature of 65 °C^40^. Similarly, Niju et al. (2016) showed high activity with a biodiesel yield of around 94% using 7 wt% of hydrated white bivalve clam shell derived catalyst with the methanol to oil molar ratio of 12:1, reaction temperature of 65 °C, and reaction time of an hour^41^.

More investigation is still needed to search for sustainable, low-cost, and efficient catalysts, alcohols, and feedstocks for improved and viable biodiesel production. In this study, mixed methanol–ethanol and eggshell-derived calcium oxide nano-catalyst were utilized for enhanced biodiesel production from waste cooking oil at different reaction parameters. Chicken eggshell-based CaO is prepared by calcination at 900 °C which is also followed by hydration- dehydration method to enhance its catalytic activity^2,6,39,42^.

## Experimental

### Waste cooking oil sample preparation

WCO was collected from nearby restaurants originally from the mixture of palm and sunflower oil. The oil mixture was filtered to remove food particles and followed by heating at 110 °C inside an oven for about half an hour to remove water present in the WCO. Moreover, the physicochemical properties of WCO such as like density, ash content, acid value, free fatty acid (FFA) value, saponification value, kinematic viscosity, and molecular weight^43^ were estimated using standard procedures.

### Preparation of CaO nano-catalyst

**Figure 1:**
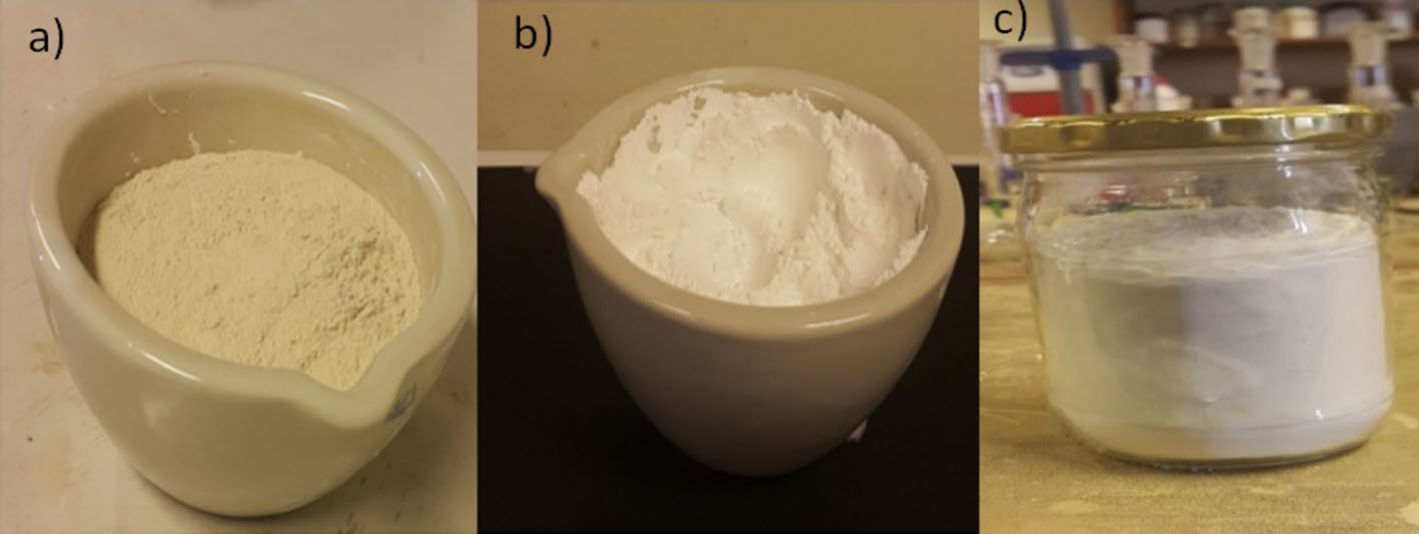
Eggshell powder (**a**) Before calcination (**b**) After calcination at 900 °C and (**c**) Calcinated eggshell after hydration-dehydration at 800 °C.

Chicken eggshell waste was collected from nearby restaurants and soaked in boiled water for 10-min to solidify the gelatinous materials adhering to the inner wall of the eggshell for easy removal. It was then washed repeatedly with tap water and rinsed with distilled water to remove impurities and followed by oven-dried at 105 °C for 24 h. After drying it was grounded and sieved by 63 µm sieve. As it can be seen in Fig. 1, the grounded eggshell powder is taken into a crucible and calcinated in a muffle furnace at 900 °C for 3 h. The calcinated eggshell powder was refluxed in the water at 60 °C for 6 h which was followed by oven dried at 105 °C over-night^41,44^.The powder was further dehydrated by calcination at 800 °C for 3 h to convert the hydroxide form into a highly porous calcium oxide nano-particle.

### Characterization of catalyst

The two different samples of CaO (the one prepared by the method of calcination of eggshell at a temperature of 900 °C and the other which undergoes further hydration-dehydration process) were characterized using X-Ray Diffraction (XRD) to determine the crystal structure and size. The XRD analysis of the sample was conducted on a diffractometer with Ni filtered CuKα radiation at λ = 0.154 nm in the range of 2 theta 5–50°. Scanning electron microscopy (SEM) (FEI INSPECT 50) and electron dispersive X-ray spectroscopy (EDX) were used to determine the morphological structure and Elemental composition of the prepared CaO nano-catalyst. The XRD results were analyzed by using origin software and the average crystal size of the catalyst was determined using the Debye Scherrer equation^45^.

### Transesterification of waste cooking oil

The transesterification process was carried out in a three‐necked 500 ml round‐bottom flask immersed in a dish as a water bath to control the temperature in the hot plate. The middle neck is used to insert a water-cooled condenser and the other neck is fixed with a thermometer to control the reaction temperature. During the reaction waste cooking oil was measured and poured into a three-necked flask and it was preheated at 50 °C and the catalyst weighed and dissolved in the required amount of preheated alcohol (calcium ethoxide/methoxide) was then, added. The transesterification process was performed to obtain the maximum yield of biodiesel by varying the operating variables such as the catalyst loading, alcohol/oil molar ratio, reaction temperature, and time. The biodiesel yield was estimated using the following equation.

$$Biodiesel \;yield\;\left( {\text{\% }} \right) = \frac{Volume\; of\; biodiesel}{{Volume\; of\; waste\; cooking \;oil}} \times 100$$

In order to investigate and optimize the effect of catalyst loading, alcohol to oil molar ratio, mixing ratio of ethanol and methanol, reaction temperature, and reaction time different experimental runs were conducted. All experiments were conducted with a constant volume of waste cooking oil of 100 ml and agitation speed of 600 rpm. To investigate the effect of catalyst loading, in each run catalyst loading varied in the range 1–4 wt% (the mass of catalyst was determined based on the weight of waste cooking oil) and the other parameters were kept constant. The initial parameter values for oil to alcohol molar ratio of 1:9, the temperature of 60 °C, and reaction time of 3 h were taken from the literature^46^.

In the next stage of determination, catalyst loading was kept constant throughout this experiment. To determine the effect of oil to alcohol molar ratio the experiment was carried out by keeping constant reaction temperature, reaction time, and previously determined catalyst loading. Oil to alcohol molar ratio was varied as 1:6, 1:8, 1:10, 1:12, 1:14, and 1:16, based on the highest biodiesel yield the value of oil to alcohol ratio was selected. A similar procedure was carried to determine the mixed ratio of methanol to ethanol by varying the ratios of alcohols as 2:10, 4:8, 6:6, and 8:4 with constant oil to alcohol ratio of 1:12 which is optimized using methanol. The reaction temperature was optimized by varying the temperature to 50, 55, 60, and 65 °C. The reaction temperature with highest biodiesel yield was taken as the optimum value.

**Figure 2:**
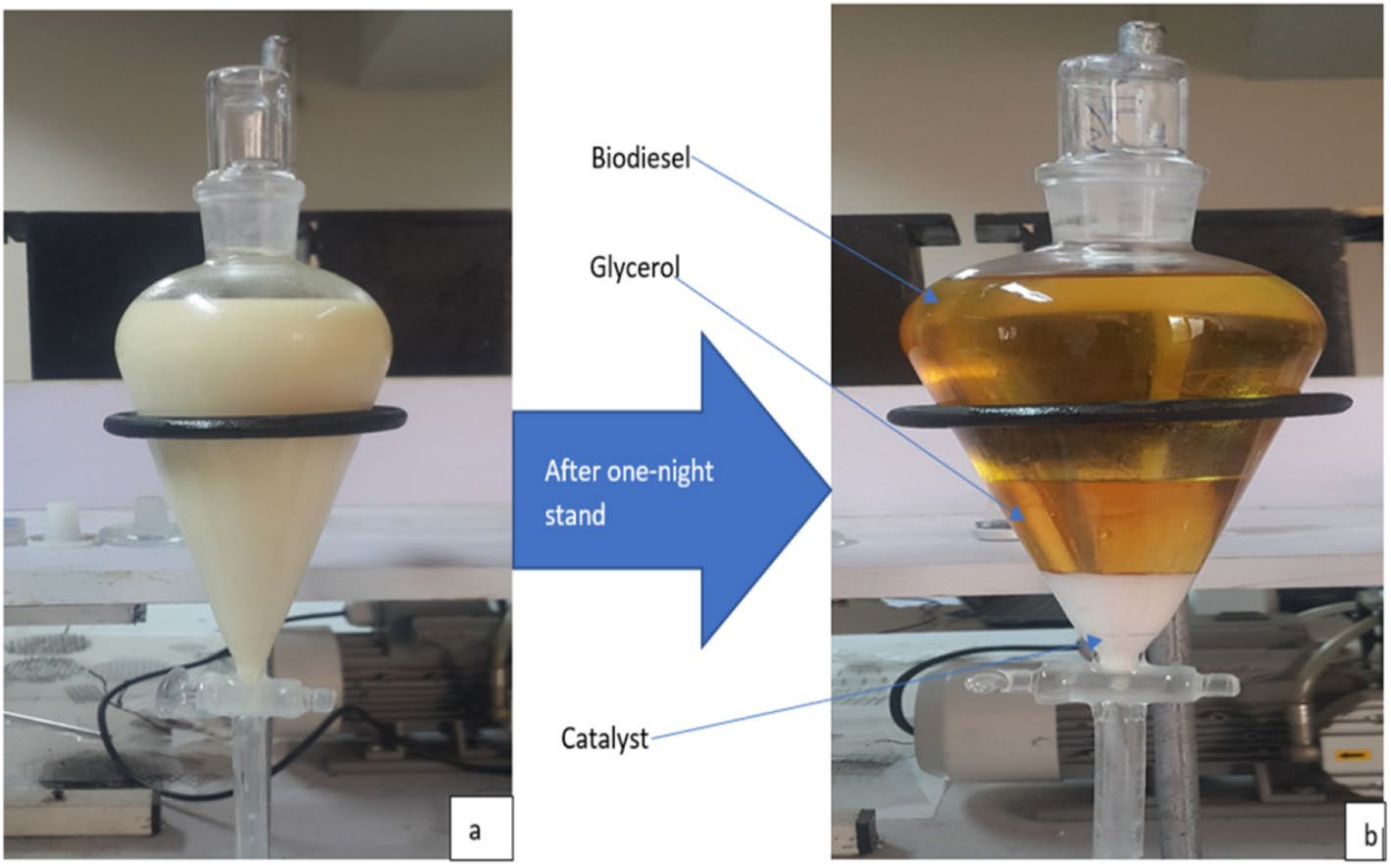
Biodiesel separation (**a**) After reaction completed before separation of glycerol, biodiesel and catalyst phase (**b**) After one-night stand glycerol, biodiesel and catalyst phase.

Finally, to investigate the optimum reaction time with the highest biodiesel yield the three values such as catalyst loading, oil to alcohol molar ratio, reaction temperature which have been previously determined in this study were taken as constant value, and reaction time was varied as 60, 90, 120, and 180-min. After the reaction has been completed, the solution was poured into separating funnel. The separate layers of glycerol, catalyst, and methyl ester/ethyl ester were distinguished. For efficient separation, the product was kept standing for over-night. After a one-night stand, As shown in Fig. 2, after the over-night stand separation of the three phases was very clear.

## Results and discussion

### Characteristics of waste cooking oil

After filtration and dehydration, the density, kinematic viscosity, acid value, free fatty acid value, saponification value, ash content, and molecular weight of waste cooking oil were determined, obtained results are presented in Table 1.

**Table 1:** Summary of physico-chemical properties of waste cooking oil (WCO).

Properties	Unit	Measured value
Density at 20 °C, g/ml	g/ml	0.9155
Acid value	mg KOH/g oil	2
Kinematic viscosity at 40 °C	mm^2^/s	46.37
Free fatty acid value	%	1
Saponification value	mg KOH/g oil	213.6475
Molecular weight	g/mol	795

### Catalyst characterization

#### XRD analysis

**Figure 3:**
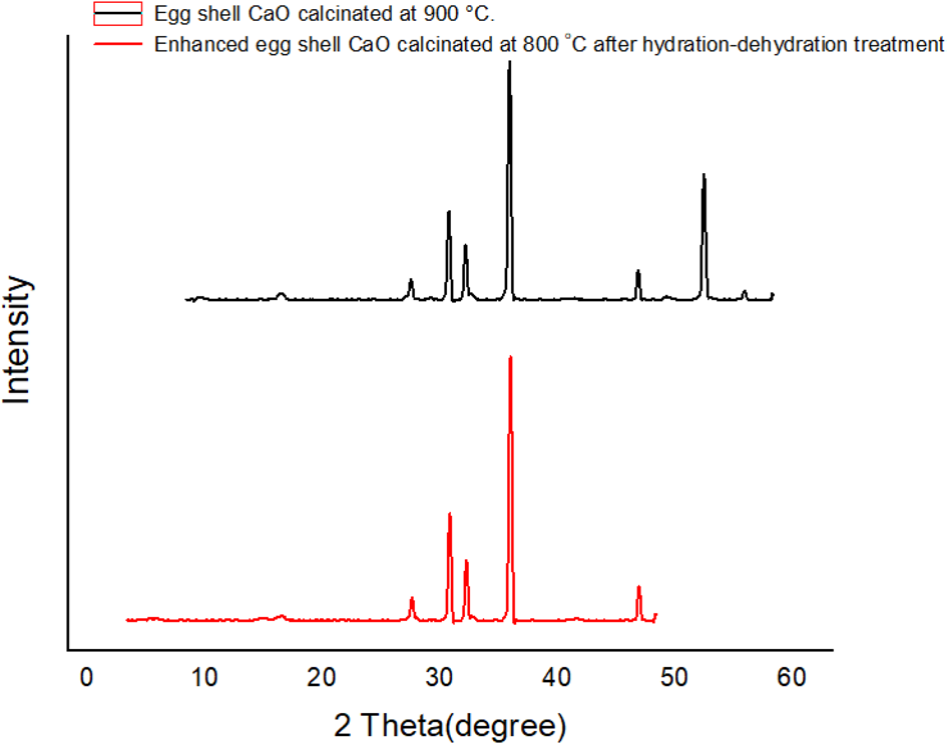
XRD pattern of CaO nano-catalyst prepared by calcination of eggshell powder at 900 °C (Black). And XRD pattern of enhanced CaO nano-catalyst prepared by calcination of eggshell powder at 800 °C after hydration-dehydration treatment (Red).

As shown in Fig. 3, the XRD diffraction pattern exhibit sharp peaks at 2-theta (2θ) values at 29.19°, 32.4°, 33.85°, 37.55°, and 48.48° for eggshell powder calcinated at 900 °C. Similarly, high-intensity peaks of 2θ values at 32.31°, 33.76°, 37.48°, 48.48°, and 54.01° were exhibited for eggshell powder prepared by calcination at 900 °C and followed by hydration-dehydration process, see Fig. 3. The presence of these sharp peaks in both materials revealed the presence of dominant CaO nanoparticles in the crystallinity of the particles. The XRD results are in a very good agreement with a similar previous report on eggshell-derived calcium oxide powder prepared from eggshell ^46^.

The calculated average crystallite size was obtained from XRD peak width analysis using the Debye Scherrer equation. Accordingly, the average crystallite sizes of 21.30 nm and 13.53 nm were obtained for eggshell-derived CaO particles prepared by calcination at 900 °C and for CaO calcinated at 800 °C followed by the hydration-dehydration method, respectively. Thus, the later catalyst preparation method provides lesser particle size, as a result, increases the surface area which in turn enhances the catalytic activity of the catalyst in biodiesel production.

The result suggests that the water treatment (hydration followed by dehydration step) has a strong effect on the crystallinity and crystalline size of the catalyst. This considerably decreases crystallinity (increase porosity) and crystalline size as a result surface area increases. This is likely associated with the evolution of water molecules, whose expulsion from the lattice during the calcination of the hydrated samples would be expected to fracture the crystallites. This phenomenon has also been demonstrated in the study by improving the transesterification activity of CaO using the hydration method. Yoosuk et al. (2010) proved that the water treatment has a strong effect on porosity, crystallinity, and crystalline size^39^. Furthermore, during hydration CaO converted to Ca (OH)_2_ then, after dehydration strong basic CaO was obtained. This is related to the fact that the basic strength and reactivity of CaO depend on its precursor. Thus, CaO obtained from Ca(OH)_2_ has strong basic characteristics than obtained from CaCO_3_^47^.

#### EDX analysis

The EDX analysis revealed that the major constituent of the eggshell-derived CaO nano-catalyst is calcium and oxygen with 54.74 and 39.76 mass percentages, respectively. The remaining trace elements in the catalyst might have not any negative impact on transesterification reaction in their oxide form rather these oxides have been reported to be active materials for transesterification^48^. The basic oxides (MgO, K_2_O, and Na_2_O) will improve the catalyst’s basic strength while the acidic components (SiO_2_, SO_3_, and P_2_O_5_) have the capacity to mediate esterification of the feedstock’s free fatty acid (FFA) content^49^.

#### SEM analysis

**Figure 4:**
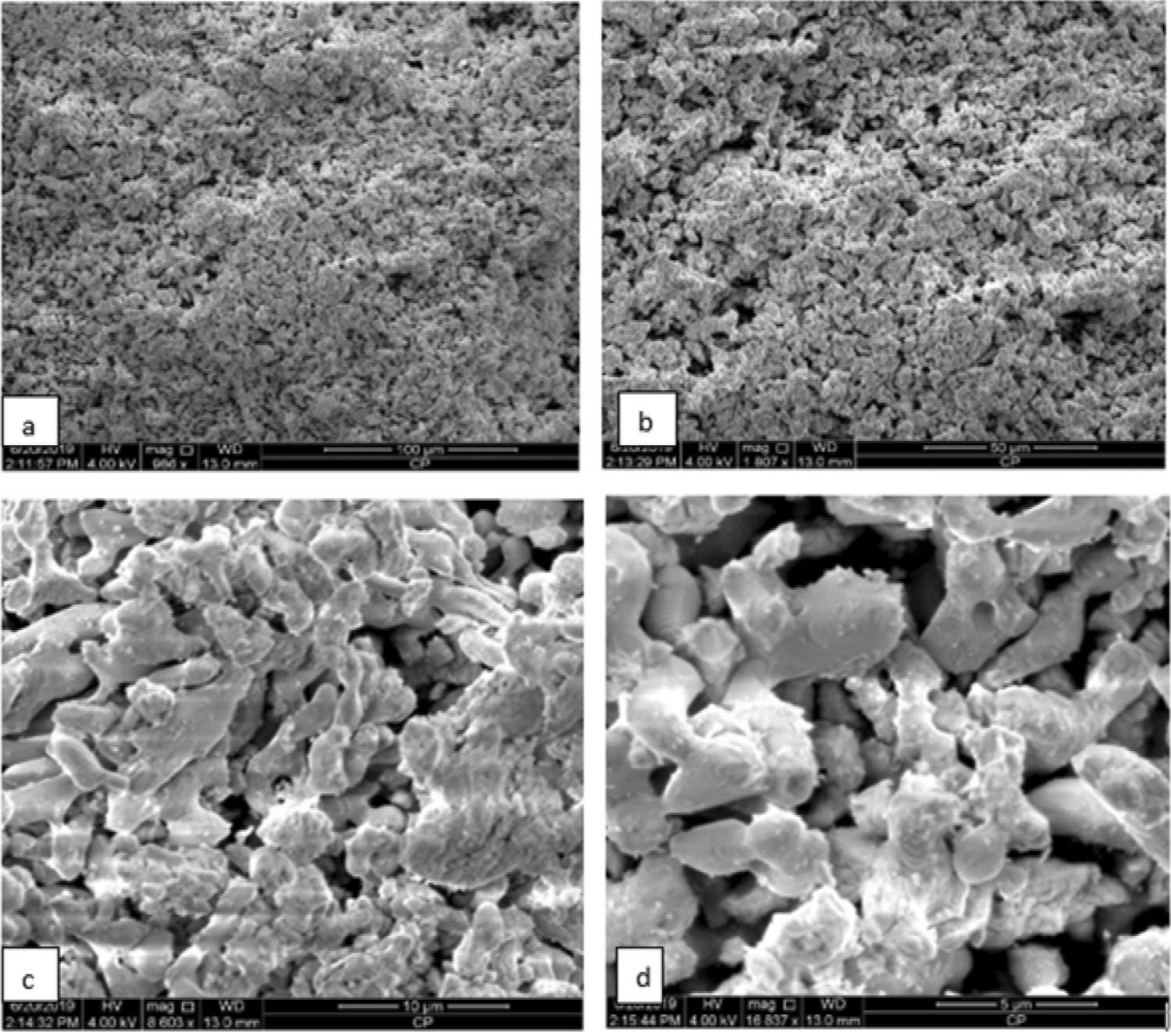
SEM image of CaO produced by calcination of eggshell at 900 °C (**a**) 100 μm, (**b**) 50 µm, (**c**) 10 µm, and (**d**) 5 µm magnification.

**Figure 5:**
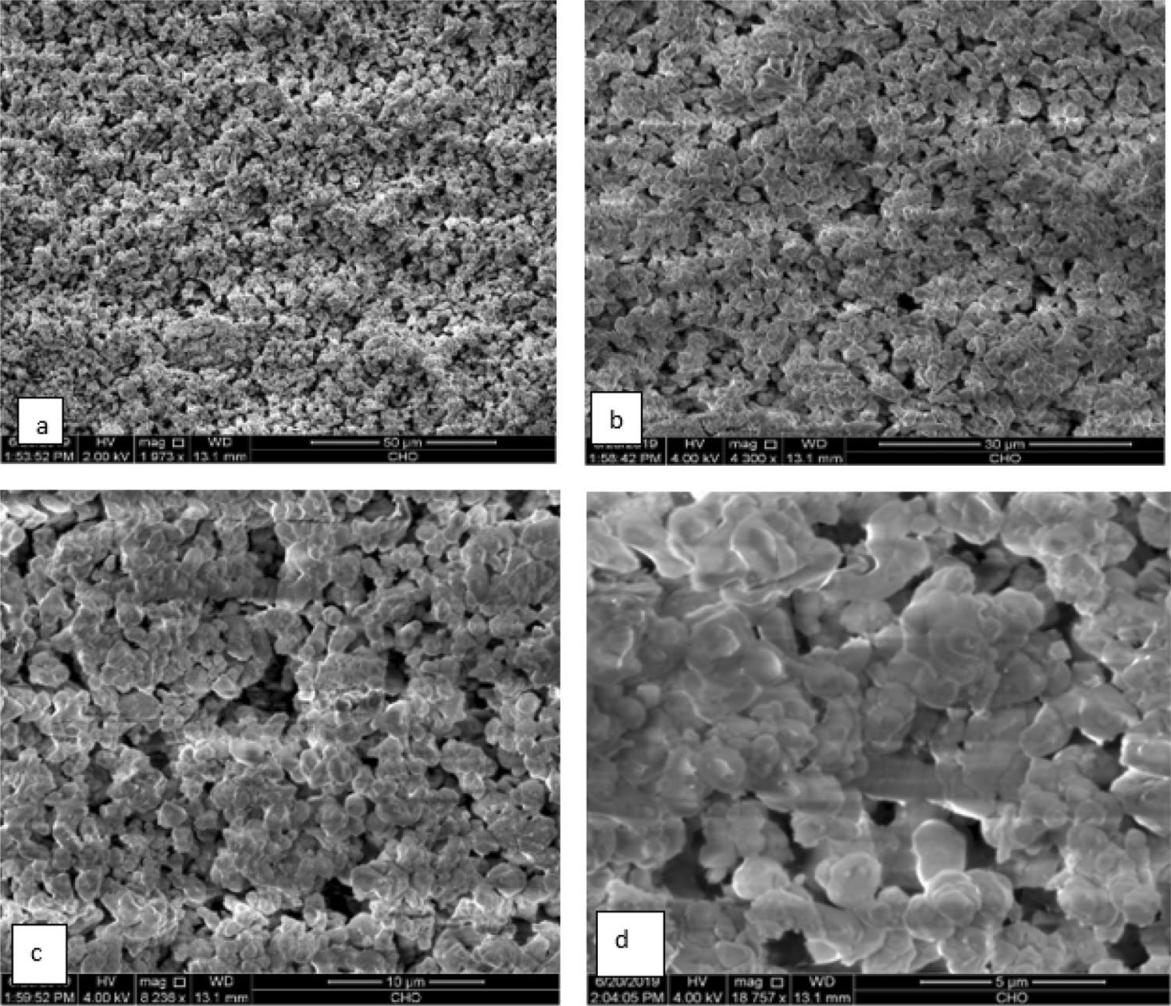
SEM image of eggshell-derived CaO produced by calcination at 900 °C followed by hydration-dehydration method (**a**) 50 µm, (**b**) 30 µm, (**c**) 10 µm, and (**d**) 5 µm magnifications.

The morphology of the obtained eggshell-derived CaO catalyst was investigated using SEM image. As shown in Fig. 4, the calcinated eggshell powder at 900 °C showed an agglomerate of rod-like regular structure. When calcinated eggshell powder was treated with water and followed by dehydration, the morphology was changed to honeycomb-like porous microstructure as shown in Fig. 5. The change in morphology after water treatment is probably due to the fact that numerous water vapor molecules is liberated from the decomposition of calcium hydroxide (Ca(OH)_2_) and the gaseous water molecules creates high porosity and more activity^42^. Besides, calcination of the catalyst after dehydration contributes to homogenize its textural properties.

### Effect of reaction parameters on biodiesel yield using methanol

#### Effect of catalyst loading

**Figure 6:**
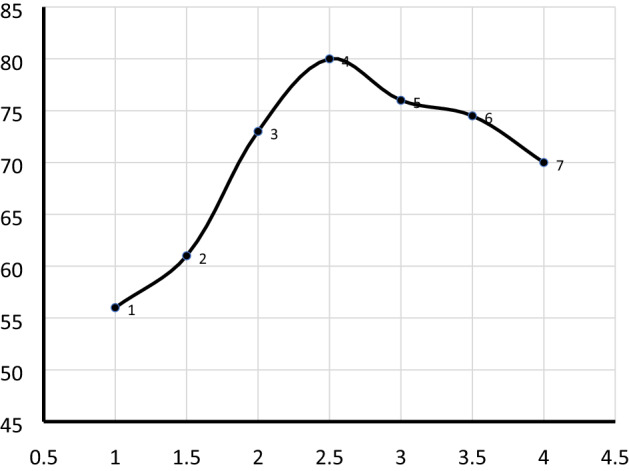
Effect of catalyst loading on biodiesel yield (%). The reaction was run at a oil to methanol ratio of 1:9, reaction time 180-min, and reaction temperature of 60 °C.

Catalyst loading is one of the key factors that determine the usefulness of the transesterification reaction. The effects of catalyst loading on the biodiesel yield are presented in Fig. 6. Based on the results, the biodiesel yield was increased with the increasing amount of catalyst loading from 1–2.5 wt%. The result indicates that increasing of catalyst loading can improve the active surface area of the catalyst that involves in transesterification reaction and enhance the biodiesel yield^26^. However, the yield was slightly decreased after 2.5 wt% up to 4 wt%. The decrement of the biodiesel after reaching optimum value can be due to larger catalyst amounts exceeding the average value makes the transesterification reaction product stickier which usually impede the mass transfer process in the liquid(oil)–liquid(alcohol)–solid(catalyst) structure. At low catalyst loading, it also deceases biodiesel yield this can be related to the amount of catalyst that is insufficient for full conversion and formation of methyl ester. In this study, the maximum yield of 80% biodiesel was obtained at an optimum value of 2.5 wt% catalyst loading.

#### Effect of oil to methanol molar ratio

**Figure 7:**
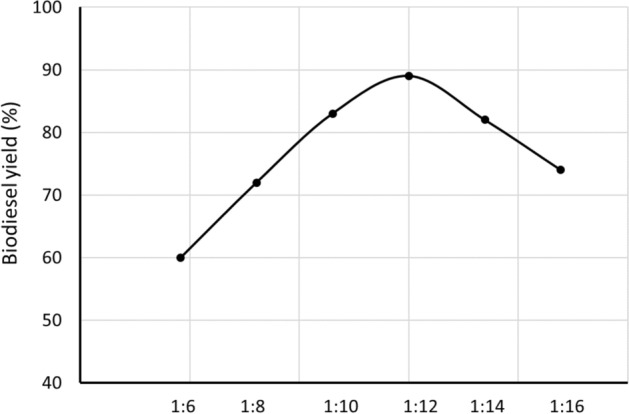
Effect of oil to methanol molar ratio on biodiesel yield (%). The reaction was run at a catalyst loading 2.5 wt%, reaction time 180-min, and reaction temperature 60 °C.

Oil to methanol molar ratio has a significant impact on the biodiesel yield^12^. As can be seen in Fig. 7, as the molar ratio of oil to methanol increase from 1:6 to 1:12 the percentage biodiesel yields also increased. Above the 1:12 ratio the biodiesel yield decreases which can be related to the high molar ratio of oil to methanol interferes with the separation of glycerin because there is an increase in solubility of glycerol in excess methanol. When glycerin remains in solution it helps drive the equilibrium back to the left, lowering the yield of esters and promoting the displacement of the balance in the opposite direction toward the formation of mono, di, and triglycerides thereby decreasing the production of esters. The overloading of methanol above optimum value would inactivate the catalyst and therefore favor the backward reaction of the transesterification process, similar effect also observed for alcohol^48^. As shown in Fig. 7, biodiesel yield decreases with an increase in the oil to methanol ratio beyond optimum value. In this study, the optimum molar ratio of oil to methanol was found to be 1:12 for a maximum biodiesel yield of 89%.

#### Effect of reaction temperature

**Figure 8:**
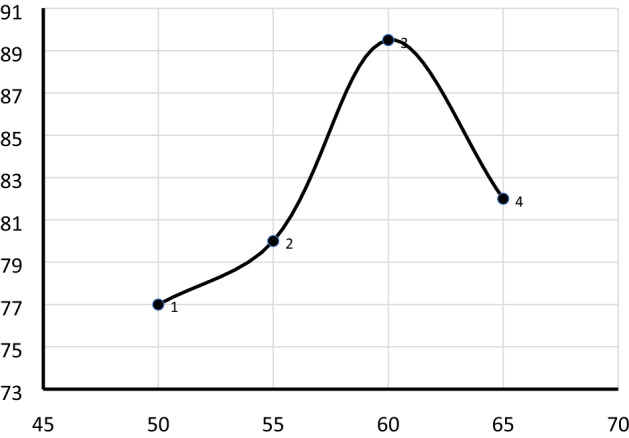
Effect of reaction temperature on biodiesel yield (%). The transesterification reaction was performed at a catalyst loading 2.5 wt%, oil to methanol molar ratio 1:12, and reaction time of 180-min.

The temperature has a significant effect on biodiesel production. As shown in Fig. 8, a maximum yield of biodiesel was obtained at 60 °C. Increasing the temperature above 60 °C resulted in a decrease in the biodiesel yield due to the fact that continuous vaporization of methanol hence methanol remained in the gas phase in the reflux, causing a decrease of methanol in the reaction media^3^. The highest yield of biodiesel was obtained at the optimum temperature of 60 °C which gave a maximum yield of 89.5%.

#### Effect of reaction time

**Figure 9:**
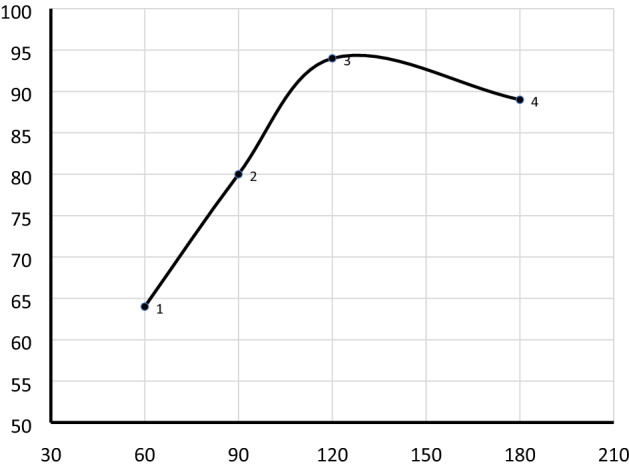
Effect of reaction time on biodiesel yield (%). The reaction was performed at a catalyst loading 2.5 wt%, oil to methanol molar ratio 1:12, and reaction temperature 60 °C.

The effect of reaction time on the transesterification process is presented in Fig. 9. The maximum biodiesel yield was attained at a 2 h reaction time. After the optimum reaction time, the biodiesel yield slightly decreases which can be due to the fact that longer reaction time leads to the reduction of biodiesel yield because of the reversible reaction of transesterification causing in loss of product. Longer reaction time may also result in the hydrolysis of esters and formed additional fatty acids to make soap consequently it reduces the biodiesel yield^50^.

Eggshell-based CaO nano-catalyst enhanced by hydration and dehydration method was used and the transesterification of waste cooking oil was carried out. The optimum value of catalyst loading 2.5 wt%, oil to methanol ratio 1:12, reaction temperature 60 °C, and reaction time 2 h was determined, and the biodiesel yield of 94% achieved. Many studies have reported the production of biodiesel by using waste shell-derived CaO. However, the catalytic performances are still lower^51^. Although many authors have reported successful transesterification reaction catalyzed by CaO, the reaction rate was slow and thus required a longer reaction time of at least 3–8 h (reaction temperature of 60–65 °C) to complete. Higher amount of catalyst and methanol is also required which resulted in high biodiesel production cost^26^. The reason behind on waste shell derived CaO required longer reaction time in order to reach high triglycerides conversion to biodiesel is due to lower basicity and surface area of waste shell derived catalyst which is known to be the major problem in heterogenous catalyst. When compared with present study the effect of enhanced eggshell based CaO on the rate of transesterification reaction is very high this is due to the improvement of the activity of CaO with hydration and dehydration technique. The effectiveness of water treatment on improving the catalytic activity of CaO has also been proved in literature^51^ by showing the hydration treated clam shell derived CaO could convert palm oil to biodiesel with approximately 98% yield after 2 h of reaction time with molar ratio of 9:1 methanol to oil, catalyst amount of 1 wt% and temperature of 65 °C.

### Effect of reaction parameters on biodiesel yield using methanol–ethanol mixture

#### Effect of oil to mixed methanol–ethanol molar ratio

**Figure 10:**
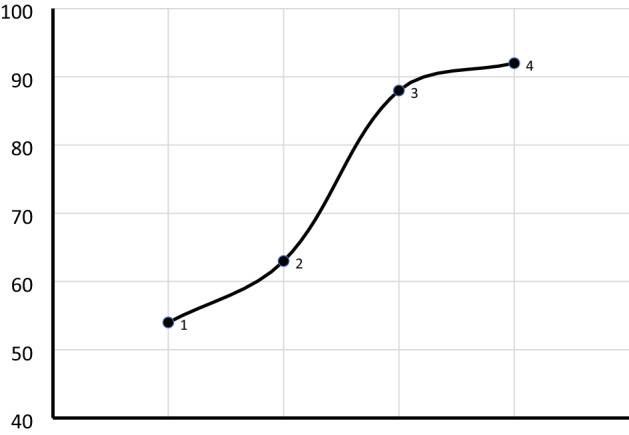
Effect of oil to methanol–ethanol molar ratio on biodiesel yield (%) performed at a catalyst loading 2.5 wt%, temperature 60 °C, reaction time 120-min, and 1:12 oil to alcohol ratio.

Mixing ethanol with methanol is one of the key ways to improve the limitation of ethanol in biodiesel production due to difficulty in product separation after transesterification. As can be seen in Fig. 10, at 2:10 and 4:8 methanol to ethanol molar ratios the separation of glycerol from the biodiesel layer was very difficult and requires a long waiting time to separate, as a result, the obtained yield was low 54% and 63%, respectively. The result indicates that domination in the amount of ethanol in the reaction causes emulsification and complicate biodiesel separation after transesterification and causing decreasing in the biodiesel yield^21^. Nevertheless, when equimolar methanol–ethanol mixture 6:6 and 8:4 ratios were used in the transesterification the separation of glycerol and biodiesel layer became easier and as a consequence, the yield significantly increased and was found to be 88% and 92%, respectively. The increase in the biodiesel yield is mainly related to the decrease in the amount of ethanol in the reaction mixture. The high biodiesel yield obtained of biodiesel at the ratio of 6:6 and 8:4 can be attributed to a combined effect of the high reactivity of methanol and better solubility of ethanol. The solubility of ethanol in oil is better than methanol and thus minimizes the mass transfer limitation between oil, alcohol, and heterogeneous catalyst. Likewise, methanol lessening the emulsification effect of ethanol and able to breakdown emulsion faster to enhance the formation of biodiesel and glycerol. Therefore, the mixed effect of methanol and ethanol was observed in this study and the study is in agreement with the previous reports^21^. The result suggests the potential replacement of methanol alcohol with renewable product bioethanol as a transesterification agent.

#### Effect of catalyst loading

**Figure 11:**
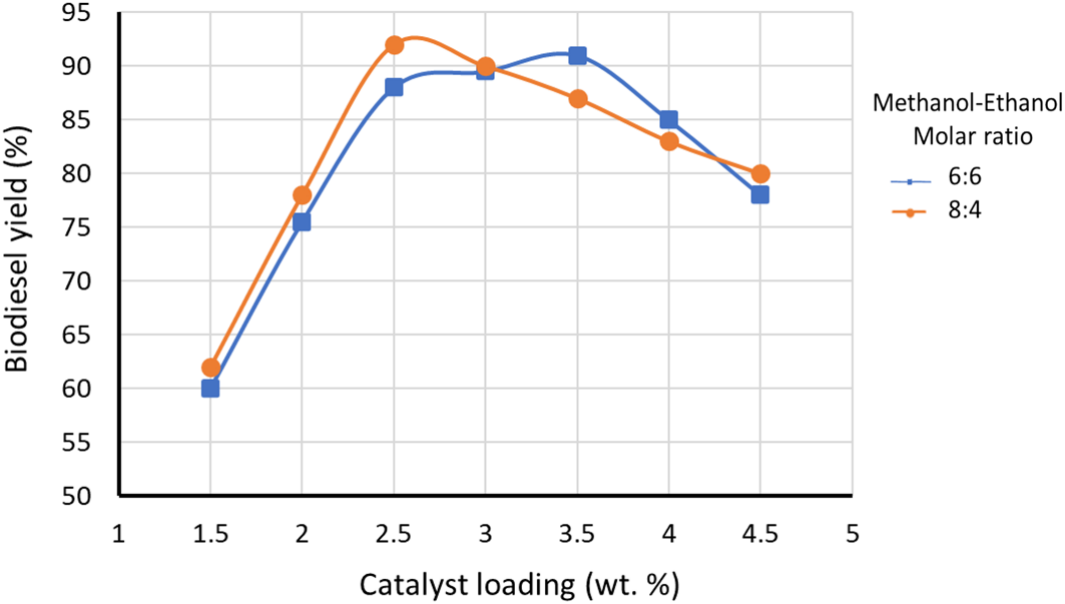
Effect of catalyst loading on biodiesel yield (%). The transesterification reaction was performed at a methanol to ethanol molar ratio 6:6 and 8:4, reaction time 120-min, and reaction temperature 60 °C.

As shown in Fig. 11, the amount of catalyst loading is varied at a different mixed ratio of methanol and ethanol. 91% biodiesel yield was obtained with mixed methanol–ethanol ratio of 6:6 using 3.5 wt% catalyst loading. However, in the case of 8:4, the highest biodiesel yield of 92% was obtained with relatively less catalyst loading 2.5 wt%. It is can be concluded from the results that when the amount of ethanol in the mixture increases, the catalyst loading slightly increases. This can be related to the high solubility of ethanol causing emulsion and as a result, relatively increase the catalyst consumption and decreasing biodiesel yield. When the alcohol mixture dominated by methanol (8:4) the maximum biodiesel obtained was relatively high (92%) with lower catalyst loading (2.5 wt%) compared to 6:6 with biodiesel yield of 88% obtained using 2.5 wt% catalyst loading. Moreover, with further increase in the amounts of catalyst, the reaction with dominant ethanol is more sensitive and the yield decreases rapidly after reaching the optimum value of catalyst (Fig. 11). This can be due to the formation of an emulsion and the viscosity which at the same time makes the biodiesel and glycerin separation very difficult^49^.

### Characteristics of produced biodiesel

Fuel properties of obtained biodiesel produced by CaO nano-catalyst synthesized from calcination and hydration-dehydration method at optimal reaction conditions were evaluated along with the American Society for Testing and Material method (ASTM). As can be seen in Table 2, biodiesel produced from methanol alone and also methanol–ethanol mixture both processes showed high-quality fuel properties within the range of biodiesel standard also in good agreement with the previous reports^52^.

**Table 2:** Fuel properties of biodiesel obtained from transesterification process using enhanced CaO prepared from calcination followed by hydration–dehydration treatment at optimal reaction conditions.

No	Property	ASTM method	ASTM limit for B 100	Biodiesel using	
				Methanol	Methanol–ethanol
1	Density at 15 °C kg/m^3^	D4052	880	874	877
2	Density at 20 °C kg/m^3^	D4052	880	859	863
3	Flash point °C	D93	100–170	105	118
4	Cloud point °C	D2500	− 3–12	9	9
5	Kinematic viscosity at 40 °C mm^2^/s	D445	1.9–6.0	4.9	5.5
6	Acid value	D974	0.50	0.45	0.48
7	Water and sediment % (v/v)	D2709	0.050	0.040	0.050
8	Copper strip corrosion, 3 h at100 °C	D130	3 max	1	1

## Conclusion

Biodiesel was produced from low-cost waste cooking oil using an enhanced CaO nano-catalyst prepared from chicken eggshell using hydration-dehydration treatment followed by calcination. SEM results revealed the change in morphology from a rod-like to a honeycomb-like porous surface with a larger surface area. Moreover, the XRD analysis disclosed a reduction in the average crystalline particle size of a catalyst from 21.3 to 13.53 nm. Hence, the additional water treatment introduced to the catalyst preparation method substantially improved the biodiesel production from waste cooking oil with mixed methanol–ethanol by enhancing its catalytic activity. As a result, a biodiesel yield of 94% was achieved under the reaction conditions of 1:12 oil to methanol molar ratio, 2.5 wt% catalyst loading, 60 °C, and 120 min reaction time. Moreover, 91% of biodiesel yield was achieved when a mixture of alcohols (i.e., ethanol-methanol), was used in the transesterification process. The ratio of methanol to ethanol affects the yield and product separation, the 6:6 and 8:4 ratios have been found to be ideal to produce high-yield with reasonable quality. The use of waste cooking oil, waste eggshell catalyst, and a mixture of methanol–ethanol in biodiesel production is relevant from the economic and environmental point of view. Hence, we can conclude that ethanol, produced from a renewable source, can feasibly substitute the common alcohol, methanol, at a sensible portion in the transesterification of waste cooking oil using enhanced CaO nano-catalyst.
